# Central resources of variant discovery and annotation and its role in precision medicine

**DOI:** 10.2478/abm-2022-0032

**Published:** 2023-08-01

**Authors:** Hashim Halim-Fikri, Sharifah-Nany Rahayu-Karmilla Syed-Hassan, Wan-Khairunnisa Wan-Juhari, Mat Ghani Siti Nor Assyuhada, Yetti Hernaningsih, Narazah Mohd Yusoff, Amir Feisal Merican, Bin Alwi Zilfalil

**Affiliations:** Malaysian Node of the Human Variome Project, School of Medical Sciences, Universiti Sains Malaysia, Kelantan 16150, Malaysia; Human Genome Centre, School of Medical Sciences, Universiti Sains Malaysia, Kelantan 16150, Malaysia; Department of Clinical Pathology, Faculty of Medicine Universitas Airlangga, Dr. Soetomo Academic General Hospital, Surabaya, Indonesia; Clinical Diagnostic Laboratory, Advanced Medical and Dental Institute, Universiti Sains Malaysia, Penang 13200, Malaysia; Institute of Biological Sciences, Faculty of Science, University of Malaya, Kuala Lumpur 50603, Malaysia; Center of Research for Computational Sciences and Informatics in Biology, Bio Industry, Environment, Agriculture and Healthcare (CRYSTAL), University of Malaya, Kuala Lumpur 50603, Malaysia

**Keywords:** annotation, discovery, precision medicine, resources, variant

## Abstract

Rapid technological advancement in high-throughput genomics, microarray, and deep sequencing technologies has accelerated the possibility of more complex precision medicine research using large amounts of heterogeneous health-related data from patients, including genomic variants. Genomic variants can be identified and annotated based on the reference human genome either within the sequence as a whole or in a putative functional genomic element. The American College of Medical Genetics and Genomics (ACMG) and the Association for Molecular Pathology (AMP) mutually created standards and guidelines for the appraisal of proof to expand consistency and straightforwardness in clinical variation interpretations. Various efforts toward precision medicine have been facilitated by many national and international public databases that classify and annotate genomic variation. In the present study, several resources are highlighted with recognition and data spreading of clinically important genetic variations.

Over the last 50 years, genetics has evolved in line with genomic technologies such as DNA sequencing [1], and currently possesses manifold applications, particularly in avenues of research where discoveries and continuously updated information pertaining to human diseases’ genome variations is useful. This then led to the rapid and high-throughput generation of sequence data, which enabled the compilation of genetics information on a bigger scale, thereby resulting in massive consequences in terms of interpretation and correlation.

With the development of next-generation sequencing (NGS), researchers can easily identify the genetic sequences in the human genome [2]. This fundamental and landmark discovery provided valuable information for understanding various functions of genes or functional genetics as well as their role in human diseases.

Genome annotation is the process of identifying functional elements in the sequence of a genome. Based on the characteristics of genomics and genetics, the variants can be categorized or prioritized for further research. The genome that has been sequenced is not completed without an annotation process, which will help determine its clinical consequences. This information is fundamental for the practice of personalized medicine. Therefore, it is important to create a convergence between human genetics, molecular biology, biochemistry, and bioinformatics in such a way that the combination of their knowledge-pools enables arriving at an understanding over how variations of DNA exist in traits and diseases [3].

Human DNA consists of single nucleotide polymorphisms (SNP), mutations, and copy number variants (CNVs). These variants constitute a cause for the differentiation of individuals based on their unique traits, including disease susceptibility and pharmacogenomics. The understanding of our genome is not only useful for the geneticist but also relevant to those working on tools and health development [4].

The 2022 study of Samuels et al. [5] is a recent development in the literature that highlights empirically based annotation techniques, together with discussing their strengths and weaknesses. Further, the study of Hebbar and Sowmya [6] focuses on various tools and methods developed for variant annotation along with their datasets.

In this review, we discuss the features, strengths, and weaknesses of various resources as well as the establishment of a genomics variants database, i.e., the Malaysian Node of the Human Variome Project Database (MyHVPDb) and countries in Asia. We hope that this review will be useful for low and middle-income countries (LMIC) in establishing their own database for their population. This review also highlights how genomic variants are classified.

There are a few resources presently available that provide the basic information that is required in genetic annotation [4]. As of today, there are approximately 147,500 data entries pertaining to locus-specific databases curating genetic variants on specific genes or diseases. However, there are differences in the types of data and nomenclature (https://grenada.lumc.nl/LSDB_list/lsdbs).

The National Center for Biotechnology Information (NCBI) has developed a database, ClinVar (http://www.ncbi.nlm.nih.gov/clinvar/), to provide information about the relationships between human variants and phenotypes, and this information predominantly assumes the form of clinical interpretation and supporting evidence [7]. In addition to these international genomic databases, the Malaysian Node of the Human Variome Project (MyHVP) was established to collate the genomics variations of different ethnicities in Malaysia, including disease variants. Despite various resources and databases that have been established, there still remain changes and limitations pertaining to these databases.

## Reference genome

Locus Reference Genomic (LRG) (URL: http://www.lrg-sequence.org) was established to curate the variants with clinical implications and contains stable genomic DNA sequences, transcript, and protein reference sequences for reporting clinically relevant sequence variants [8]. The updatable section contains the mapping of the LRG to the most recent genome build and the most advanced and up-to-date biological knowledge for the LRG region from Ensembl and NCBI. To ensure that each LRG record is updated, the curators review all existing LRGs twice a year and when a new genome build is released.

The LRG is aligned to the reference genome to determine its mapping coordinates. Each area of contiguous alignment is described separately, along with any differences between the LRG and the reference. The existence of LRG is important for researchers to understand the function of a gene and how variations might affect its function and cause clinical implications [2]. The reference genome is not from a single person only. It is derived from the DNA of certain people, as data sequenced from thousands of genomes will show a drastic variation in DNA content. Among the advantages of having a reference is that the genome would be useful in analyses employing DNA testing for forensic purposes, as well as paternity determination, through the repetitive sequence of variant regions.

## Importance of variant annotation and interpretation

An individual's DNA varying from those of other individuals is caused by differences in ancestral pattern, unique traits, and susceptibility to certain diseases [9]. Therefore, a greater understanding of variant annotation and interpretation is essential and contributes significant roles to further determining how this part of the genome influences life and how knowledge of these variants can be applied toward optimal health management and care [3].

Variant annotation is an important step for the precise determination of sequence variants, as genetic alterations may result in various effects on genes and proteins, including a gain of function mutation that commonly affects the protein by introducing new protein functions such as missense mutation. Additionally, genetic alteration may also be caused by loss of function mutation, which could abolish the protein function. The effect may result in nonsense mutation that terminates the proteins or splice site mutation that can disrupt RNA splicing, causing alteration to protein sequence. By annotating the variants according to their functional classification, it would be easier for researchers to prioritize their target variants and perform further interpretation.

## Next-generation genome annotation and the challenges

Curation of the human genome is a time-consuming process because it depends on unstructured sources such as published literature, collections of laboratory clinical data, disease-specific groups, and databases of functional experiments. While the genome sequencing revolution has led to the sequencing and assembly of many thousands of new genomes, genome annotation still uses very nearly the same technology that we have used for the past 2 decades. Variant identification and annotation are pivotal starting points for an accurate variant interpretation. The prevailing challenge in the variant annotation is the conversion of genomic variant coordinates to their corresponding localization of cDNA or amino acid [10]. Genome interpretation is highly dependent on the well-defined genomic variant coordinates, as the difference in the alignment of sequence variants may lead to incorrect variant nomenclature. An accurate variant interpretation is also determined by a consistent variant representation using a correct transcript accession [10].

Additionally, issues and arguments of data inconsistency may arise due to disparities between the laboratories and consequently cause difficulties for clinicians in fully utilizing genetic information for their patients’ management. To successfully resolve these discrepancies, broad, up-to-date, and flexible standards that are best equipped and in line with the expanding genomics information should be deployed, so as to ensure the benefit of overcoming any argument in clinical classifications [11, 12]. Additionally, various variant detection software tools can strictly identify a specific alteration, including SNV, indels, structural variants, and CNV. Clinical laboratories must also understand and be aware of any limitations of the variant detection tools. The quality of the results from each laboratory should be maintained through an appropriate laboratory validation of the bioinformatics pipeline and the use of commercially purchased bioinformatics tools and software [11, 12].

Another critical point to be considered is the inclusion of significant clinical information and interpretation of the identified variants such as the gene symbol, the location of the variant, type of variant, correct HGVS nomenclature, and predicted protein sequence alterations. An additional source of information, such as cross-references to cancer-specific databases, multiple genomic databases, population frequency databases, and pre-computed in silico-based prediction tools, is also very useful in assisting in variant interpretation [10].

Variant annotation is greatly dependent on computational predictions to characterize the effect of the variant on a molecular level. While the use of this prediction tool was aided by the availability of published reports and an expert panel for validation, few limitations still exist in these tools. For annotation tools such as SIFT and PolyPhen that predict the functional effect of SNP on protein and phenotype, these tools have their own limitations. SIFT could not predict the protein structure while PolyPhen includes that information; however, the accuracy of this tool depends on the structural class of the protein [3, 13]. Various databases have now provided gene expression data in addition to genome annotation and allele frequencies for population-based information [14, 15]. However, these databases are still characterized by a few limitations, including an absence of data integration for different kinds of information, and an example of this particular limitation would be that, for a given DNA variant query at any one time, gene expression data are not available in a state of incorporation within the functional prediction and pathogenicity classification [16]. Most of these databases also only allow a limited number of gene and DNA variant queries [17]. Massive data for variant interpretation has been a major challenge for researchers and the integration of different information from the available resources to support the evaluation of evidence for data interpretation will be a better solution to resolve this limitation. At this point, variant annotation from multiple annotation tools and databases must be considered for a complete variant interpretation [18].

## Global initiatives on variant annotation

Selected resources, as described in **Table 1**, show the valuable information and analysis tools needed to portray genetic variations along the proof continuum [19]. These public resources consist of variation data from both normal and affected populations [20]. Advancements in innovation and investigation abilities, resulting in costs’ and time consumption reduction, have alternately changed genomics, empowering one to pinpoint applicable genetic variants inside a large number of variations in a sequenced database [21]. However, recovering factual and useful annotation important at the single nucleotide level is still problematic [22].

**Table 1.** Selected resources, valuable information, and analysis tools available for variant annotation

**Resource**		**Brief description**	**Features**	**References**
**Database**				
1	dbVar	dbVar is an archive of large-scale genomic variants (generally >50 bp) including insertions, deletions, duplications, inversions, mobile elements, translocations, and complex variants. The current number of submitted variants in the database is more than 6 million from over 190 studies including large international projects such as 1000 Genome Project, gnomAD, the CNV Global Population Survey, and clinical resources, which are ClinVar and ClinGen.	To date, the total in the dbVar database of 190 studies consists of 6 million regions and 36 million variants. dbVar continues to make it easier to find and use structural variation data by making selected datasets available on TrackHub for viewing in the NCBI GDV and other genome browsers, such as the UCSC browser. Tracks are available for the structural variants imported from ClinVar (https://www.ncbi.nlm.nih.gov/dbvar/content/clinvar_summary/#homepage) and for the NCBI Curated Common Structural Variants (nstd186).	[23, 24, 25]
2	HGMD	A thorough assortment of published germline mutations in nuclear genes that entail, or are firmly connected with, human acquired disease.	HGMD has been supported over the years by commercial partnerships with various industry leading biomedical research companies. A stand-alone web application has been made available under license from BIOBASE GmbH. The latest version of HGMD (2020.2) contains 289,346 different mutations in 11,076 genes (more than ClinVar & OMIM). Articles identified as potential sources of mutation data are assessed by a team of experienced curators (with an average of more than 12 years’ experience in curation). Academic or non-profit users without a subscription may utilize the public version of HGMD (http://www.hgmd.org). However, this version is provided in a basic form that is searchable only by gene symbol or disease name, is only updated twice annually, is maintained permanently at least 3 years out of date, and does not contain any of the additional annotations or extra features present in HGMD Professional. Another challenge is that an increasing number of journals do not appear to be systematically indexed by Medline, at least not immediately upon publication.	[26] http://www.hgmd.cf.ac.uk/ac/index.php
3	LOVD	The purpose of LOVD is to provide a flexible and freely available tool for genomic variant and phenotype collection, display, and curation.	LOVD allows both patient-centered and gene-centered views as it is open sourced and released under the GPL license. LOVD is actively being improved. Using Leiden server, LOVD offers free hosting and support of LOVD-powered gene variant databases.	[27, 28] https://www.lovd.nl/3.0/publiclist
4	gnomAD	An asset created by a global alliance of specialists, with the objective of amassing and blending both exome and genome sequencing information from a wide assortment of huge scope sequencing researches and making rundown information accessible for the more extensive academic local area.	The data released by gnomAD are available free of restrictions and proved to be fertile ground for testing new approaches to variant interpretation. Major limitations on analysis is the quality and size of the available reference databases of normal genetic variation and the data is only focused entirely on small variants.	http://gnomad.broadinstitute.org
5	dbSNP	An information base of short genetic variants that list variations with population-genetic annotations, file germline variants for both rare mutation and polymorphism, and give alleles, genotypes, and their individual frequencies based on their population.	Data within dbSNP are available freely and in a variety of forms. There is no requirement or assumption about minimum allele frequencies or functional neutrality for the polymorphisms in the database. The validation code in the SNP record provides some limited information on how the SNP was identified and on the experimental evidence confirming its existence. The quality of the data found on dbSNP has been questioned by many research groups, which suspect high false positive rates due to genotyping and base-calling errors.	[29] http://www.ncbi.nlm.nih.gov/snp
6	GenBank	A public database that contains 9.9 trillion base pairs from over 2.1 billion nucleotide sequences for 478,000 formally described species.	A public database in which nucleotide sequences are available for 400,000 formally described species, and which handles large sequence records. Daily data exchange with the Europe Nucleotide Archive and the DNA Data Bank of Japan ensures worldwide coverage.	[30, 31]
7	ClinGen	Makes an assembly of organization assets to reform our perception of genomic variants and perk up its use in clinical thought with related data set in ClinVar.	The ClinGen variant curation process combines clinical, genetic, population, and functional evidence with expert review to classify variants into 1 of 5 categories, which are pathogenic, likely pathogenic, uncertain, likely benign, and benign according to the ACMG guidelines.	[32] https://clinicalgenome.org/
8	ClinVar	Totals data on variation of sequence and its association to human wellbeing and giving affirmations of variation that are of clinical significance.	In December 2019, ClinVar reached the milestone of 1 million submitted records, representing more than half a million variants. ClinVar allows a user to “follow” a particular variant and be notified if the overall clinical interpretation in ClinVar changes, for example from a pathogenic category to a non-pathogenic one. This feature makes it easier for a laboratory to become aware of variants that may need to be re-evaluated, and for clinicians to know when they should contact their clinical testing laboratory, patient, or both with new information.	[7, 23] http://www.ncbi.nlm.nih.gov/clinvar

**Tools**				

1	Phenotype Based Gene Analyzer (Phenolyzer)	An instrument zeroing in on finding genes dependent on client explicit disease or phenotype terms.	Phenolyzer includes 5 components and works based on an intuitive approach. Phenolyzer exhibits superior performance over competing methods for prioritizing Mendelian and complex disease genes, based on disease or phenotype terms entered as free text. Compared to wANNOVAR, Phenolyzer is better at prioritizing candidate genes for Mendelian diseases and complex diseases.	[33] http://phenolyzer.wglab.org/
2	VarAFT software	Provides annotation and determines human disease-causing mutations by accessing various levels of data.	It combines information from the dbNSFP and ANNOVAR, OMIM, HPO, Gene Ontology, pathways (Reactome), KEGG, PID, predictions from UMD-Predictor, and HSF. The filtration features the prioritization of candidate mutations and it is a freely available software.	[34] http://varaft.eu
3	Ensembl	A product framework of genome data sets for vertebrates and other eukaryotic species, which delivers and keeps up programed annotation on chosen eukaryotic genomes	The framework indicates the role played by variant effect prediction (VEP) “plugins” in expanding variant functionality, and the plugins considered include Missense Tolerance Ratio (MTR) and Rare Exome Variant Ensemble Learner (REVEL); additionally, the variant data integration from NCBI dbSNP and European Variation Archive (EVA) is considered, as well as the population frequency data from Genome Aggregation Database (gnomAD). It displays variant locations on experimentally derived 3D protein structures.	[35, 36, 37] http://www.ensembl.org
4	SNPnexus	An online variant annotation instrument intended to improve and aid the determination and prioritization of known and novel genomic alterations	It applies DeepSEA scoring algorithm for non-coding variants and CGI for identification of somatic alterations. Genes are linked to associated pathways using Reactome.	[38, 39]https://www.snp-nexus.org/v4/
5	UCSC genome browser	An online instrument for rapidly showing a mentioned segment of a genome at any magnitude, joined by a progression of aligned annotation “tracks”	Researchers are able to customize their annotation track based on the track hubs available in the genome browser. The framework allows a broad selection of annotation track; for example, GTEx Gene track, which is used to display gene expression levels across various tissue types; NCBI RefSeq tracks; and gnomAD, which is used for whole genome and whole exome datasets.	[40, 41, 42, 43, 44] http://www.genome.ucsc.edu/
6	COSMIC	A worldwide asset infiltrating the world literature on somatic mutations in human cancer	It provides functional descriptions of cancer genes and consists of 2 tiers of CGC to classify genes based on their strength of evidence associated to their role in cancer. A new platform of COSMIC-3D, for understanding of cancer mutations in 3D protein structure	[45, 46, 47] http://www.sanger.ac.uk/perl/genetics/CGP/cosmic
7	ITHANET	A global site devoted to thalassemia and different hemoglobinopathies, which gives free admittance to data sets and devices applicable to various parts of hemoglobinopathies, for example, sequence variations, epidemiology, and phenotype	The website provides the most recent content update with its friendly user interface and is easy to navigate. It provides multiple external links to other databases.	[48, 49] https://www.ithanet.eu/
8	Genenames.org	The HGNC based at EMBL-EBI assigns unique symbols and names to human genes.	It contains almost 42,000 approved gene symbols, over 19,000 of which represent protein-coding genes, and displays all approved nomenclature within Symbol Reports that contain data curated by HGNC editors and links to related genomic, phenotypic, and proteomic information. It is accessible via https://www.genenames.org	[50, 51, 52]

**Consortium**				

1	ICGC	The ICGC is a global initiative to build a comprehensive catalog of mutational abnormalities in the major tumor types.	ICGC's Data Portal is a user-friendly platform for efficient visualization, analysis, and interpretation of large, diverse cancer datasets. The portal currently consists of data from 84 worldwide cancer projects, collectively representing about 77 million somatic mutations and molecular data from over 20,000 contributors. The ICGC dataset comprises several high-level data types, including donor, gene, mutation, and cancer drug. To support fast, simultaneous querying of these entities and their relationships, the ICGC developed a sophisticated search framework based on the document-oriented indexing technology Elasticsearch (https://www.elastic.co/), a distributed search and analytics engine that features fast query speed, massive scalability, and flexible JSON schemas. It takes ~100 ms in a search across the 77 million ICGC mutations to retrieve the 10 most frequent mutations found in brain cancer donors and those that occur within genes cataloged in the COSMIC CGC (https://cancer.sanger.ac.uk/census).	[53]

**Project**				

1	100,000 Genome Project	The biggest worldwide coordinated effort, which has sequenced 100,000 genomes from around 85,000 NHS patients influenced by an uncommon disease, or malignant growth	The 100,000 Genomes Project aims to sequence 100,000 genomes from NHS patients with cancer and rare diseases. Data collected from the 100,000 Genomes Project can inform research on rare diseases, or benefit patient care potentially by streamlining the diagnostic process and tailoring care to the individual. The project was established to sequence 100,000 genomes from around 85,000 NHS patients affected by a rare disease, or cancer. The Project would also create a new genomic medicine service for the NHS – transforming the way people are cared for and bringing advanced diagnosis and personalized treatments to all those who need them. The 100,000 Genomes Project improves the field of molecular medicine through the improvement of diagnosis of disease, earlier detection of genetic predispositions to disease, rational drug design, gene therapy, and control systems for drugs and pharmacogenomics “custom drugs.” The Human Genome Project could help with the diagnosis and prevention of human disease, which allow to modify medication for more effective treatment cycles, improve criminal justice proceedings, helped to boost the economy and can help more than just humans. The Human Genome Project may cause a loss in human diversity, by developing a trend in “designer” humans. Its information could be used to form new biological weapons and could become the foundation of genetic racism as it would be most accessible to wealthy cultures.	[54] https://www.genomicseng-land.co.uk/about-genomics-england/the-100000-genomes-project/
2	GENCODE	A project to identify and classify all gene features in the human and mouse genomes with high accuracy based on biological evidence, and to release these annotations for the benefit of biomedical research and genome interpretation	The project annotates human and mouse genes and transcripts supported by experimental data with high accuracy, providing a foundational resource that supports genome biology and clinical genomics. GenCode annotation is accessible via Ensembl, the UCSC Genome Browser, and https://www.gencodegenes.org	[55, 56, 57]

**Guidelines**				

1	ACMG Guidelines	An established guidance for the interpretation of sequence variants.	It provides the guidelines of variant classification, namely “pathogenic,” “likely pathogenic,” “uncertain significance,” “likely benign,” and “benign,” based on criteria using typical types of variant evidence (population data, computational data, functional data, segregation data, etc.). These 5 features are applied to the ClinVar database.	[58] https://www.acmg.net/docs/standards_guidelines_for_the_interpretation_of_sequence_variants.pdf

ACMG, American College of Medical Genetics and Genomics; CGC, cancer gene census; CGI, cancer genome interpreter; COSMIC, catalogue of somatic mutations in cancer; ClinGen, Clinical Genome Resource; CNV, copy number variant; dbSNP, database for single nucleotide polymorphism; EMBL-EBI, EMBL's European Bioinformatics Institute; EVA, European Variation Archive; gnomAD, Genome Aggregation Database; GTEx, genotype-tissue expression; HGMD, Human Gene Mutation Database; HSF, human splicing finder; HGNC, HUGO Gene Nomenclature Committee; ICGC, International Cancer Genome Consortium; JSON, JavaScript Object Notation; LOVD, Leiden Open (source) Variation Database; MTR, Missense Tolerance Ratio; NCBI; National Center for Biotechnology Information; OMIM, Online Mendelian Inheritance in Man; REVEL, Rare Exome Variant Ensemble Learner; SNP, single nucleotide polymorphism; VEP, variant effect prediction.

### Initiatives by LMICs in establishing genomics variants database

Several LMICs have established their own ethnic-specific genome databases in preparation for future implementation of personalized medicine. As an example, Thailand has developed the Thai Reference Exome (T-REx) variant database (https://trex.nbt.or.th/) that can be used to filter and prioritize pathogenic candidate mutations among the Thai population [59]. T-REx is part of the Genomic Thailand Initiative, which the Thai government supports, with the aim of sequencing the genomes of 50,000 diverse individuals among the Thai population so as to establish an infrastructure for harnessing genomic information. The T-REx variant database contains information regarding short variants and CNV genotyped from 1092 unrelated populations of Thailand [59].

In the same context, Iran has genotyped 800 individuals from 8 major ethnic groups, which has contributed to establishing the Iranome database (www.iranome.com). This database contains 1,575,702 variants, of which 308,311 were novel variants (19.6%). The clinical information in this database can be a useful tool for categorizing the pathogenicity of uncommon mutations [60].

Malaysia is a middle-income country located in Southeast Asia and is a member of the Human Variome Project. Its services in medical genetics started in 1994 and are improving within 2 decades. The availability of medical genetic services includes genetic testing, diagnosis, and counseling for both adult-onset inherited and pediatric conditions [61]. Up to now, variant information of the Malaysian genome is under-represented in many databases. Therefore, with the objective of ascertaining and documenting molecular genomic variations across the Malay population, Malaysia has initiated the first database for this population, namely the Malaysian Node of the Human Variome Project (MyHVP), which includes the Chinese, Indian, and Orang Asli ethnicities in Peninsular Malaysia, as well as various minority ethnicities in Sabah and Sarawak. The MyHVPDatabase (MyHVPDb) was developed as a database containing genetic variants of both healthy and diseased Malaysians based on their ethnic groups [62].

MyHVPDb serves as a repository of information about mutations, and can have applications in future undertakings by researchers worldwide, especially those focusing on precision medicine. To date, a total of 189 genes consisting of 2571 variants are recorded in MyHVPDb, and a majority of the sources of genetic information originate from individuals belonging to the Malay, Orang Asli, Indian, and Chinese ethnic groups in Malaysia. These variants are associated with 112 diseases including beta-thalassemia, multiple endocrine neoplasia, breast cancer, and retinoblastoma. About 291,718 SNPs were genotyped from 101 healthy Malay individuals and deposited in MyHVPDb.

MyHVPDb was developed using the Leiden Open Variation Database (LOVD) version 3. LOVD is widely used for the standard variant databases system for the collection, display, and curation of DNA variants including phenotypes and diseases [63]. The current version of LOVD 3 introduced a more sophisticated patient phenotype model as an important feature for this version. This allowed phenotype information to be recorded over time as a disease is diagnosed, treated, or progresses in a patient [63]. Having the phenotype feature will fulfill our future plan to add the clinical data in the MyHVPDb as an important resource to the clinicians in the field of genetic medicine.

The Singapore Genome Variation Project (SGVP) provided a publicly available resource of 1.6 million SNPs in the past 12 years that were genotyped in 268 individuals from the Chinese, Malay, and Indian population groups in Southeast Asia [64]. This project aims to characterize genomic variation and positive natural selection in 3 major Asian population groups (Chinese, Malay, and Indian). With the genomic information that is already available, Singapore is currently working on a national strategy for developing personalized medicine, which is called Singapore's National Precision Medicine (NPM). The goal is to improve and protect the health of every Singaporean by strengthening public health, enhancing disease prevention, and discovering the most appropriate therapies for the most appropriate individuals and groups [65]. Until now, Singapore has been the leading country in developing the field of personalized medicine when compared with other countries in the Asian region. The NPM was launched in 2017 and is a 10-year project, which has already entered the second phase in April 2021. The goal is to enhance and accelerate Singapore's biomedical research, health outcomes, and economic growth [65].

In Indonesia, a developing country, approximately 2500–5000 babies are born each year with a clinical pattern of beta-thalassemia major and beta-thalassemia intermedia [66, 67]. The prevalence rate for beta-thalassemia carriers varies in the range of 3.0%–10.0% throughout regions of Indonesia [68].

In general, DNA diagnosis is not routinely performed in the health service, and diagnosis of suspected thalassemia individuals usually comprises complete blood count and hemoglobin electrophoresis [68]. Several studies have mapped several local mutations in large, multi-ethnic cities in Indonesia [66, 67]. In Indonesia, the task of collection of thalassemia patients’ information and compiling these into databases has not been implemented except on a very limited scale, since many of these patients have no allelic mutation detected. Since this patient database has still not been managed effectively, a national effort in this direction is needed, which may be initiated by a government agency dedicated especially to this task.

### Guidelines for the interpretation of variants

The American College of Medical Genetics and Genomics (ACMG) Standards and Guidelines were predominantly developed as an educational guideline for clinical laboratory geneticists, to assist them in providing a quality clinical laboratory service [58].

### ACMG Guidelines

In 2015, the American College of Medical Genetics and Genomics (ACMG) and the Association for Molecular Pathology (AMP) released joint recommendations for interpreting variants in genes associated with Mendelian disorders. These recommendations are part of an effort to establish a common framework for variant classification based on a standardized and transparent assessment of different lines of evidence. ACMG Guidelines provide several levels of evidence to ascertain the clinical significance of each genomic variant. However, there is still a challenging task to determine the pathogenicity of variants, especially in cancer. Several factors and information need to be taken into account as each genomic alteration can have an extent of much clinical efficacy, such as the diagnosis, prognosis, therapy selection, and monitoring, though only a few cancers have strong evidence of pathogenicity [69]. Until now, peer-reviewed literature, guidelines for clinical practice, and any relevant mutation database are being utilized as the main sources in equipping these variants with useful clinically significant evidence for further assessment [70].

**Figure 1.**
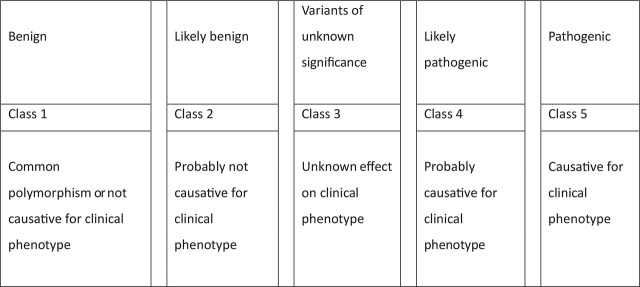
The 5 variant classifications of ACMG Guidelines [58].

The ACMG–AMP Guidelines propose the use of specific terminology, namely “pathogenic,” “likely pathogenic,” “uncertain significance,” “likely benign,” and “benign,” to characterize variants in Mendelian disorders (**Figure 1**). The variants are classified into these 5 categories according to different types of evidence including population data, computational data, functional data, and segregation data. This framework defined 28 evidence criteria organized by type and strength, which are used to classify the variants into the 5 categories. The framework proposed by the guidelines, encompassing various genes, diseases, and inheritance patterns, is intended for general use; however, to effectively execute the process of variant interpretation, expert judgment in evaluating and weighing evidence is needed.” By using the guidelines, a mutation is characterized by a change in the DNA base sequence, whereas a polymorphism is characterized by a frequency of greater than 1%. Both mutation and polymorphism were later changed to “variant” to avoid confusion arising from inaccurate assumptions of pathogenic or benign effects [58].

The Clinical Genome Resource (ClinGen) convenes all Variant Curation Expert Panels (VCEPs) to perform gene- and disease-specific modifications to the ACMG–AMP framework for variants related to a particular disease and inheritance pattern [32]. Additionally, members of ClinGen have formed a working group, called the Sequence Variant Interpretation Working Group (SVI WG). This working group aims to provide refinements of ACMG–AMP guidelines across multiple domains and to harmonize guidelines made by individual VCEPs [71]. SVI WG has published these recommendations (https://www.clinicalgenome.org/svi/).

**Figure 2.**
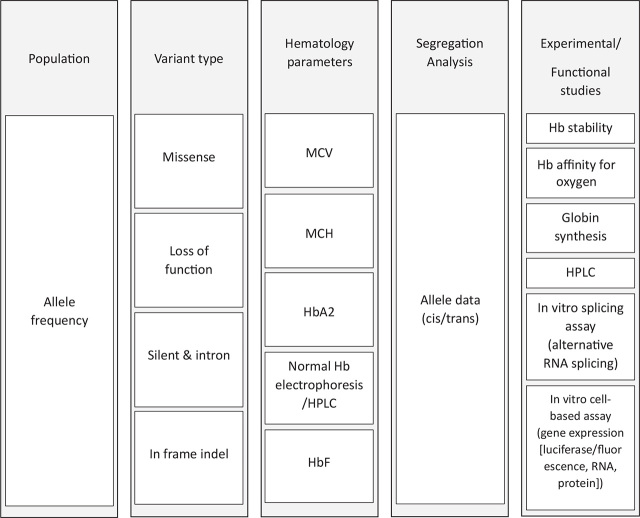
Parameters required for variant annotation in hemoglobinopathies.

The current effort of ClinGen is the development of the ClinGen Variant Curation Interface (VCI). The ClinGen VCI is a global open-source variant classification platform that helps with the use of evidence criteria and the classification of variants based on the ACMG–AMP variant classification guidelines. There are 43 VCEPs and the majority are in the process of the establishment (https://clinicalgenome.org/affiliation/vcep/#ep_table_heading). **Figure 2** indicates the parameters used by the ClinGen Hemoglobinopathy VCEP for annotation variants in disorders of hemoglobinopathies.

Although these modifiers may not fully represent human phenotypes, they comprise a 5-tier system of classification for variants that are significant to Mendelian disease, which should be supported with a clinical condition and inheritance pattern.

## Variant interpretation in precision medicine and its challenges

Precision Medicine Initiative (PMI) was initiated in 2015 to accelerate and enhance the understanding of precision medicine and to take into consideration factors such as genetics, physiology, and environmental parameters for ascertaining the best approach to disease treatment for any individual patient [72]. Previous and current medical practices usually do not consider this biomedical information due to inaccessible and insufficient sources. Additionally, genome sequencing and extensive biomedical data collection are usually still not an option due to the costs involved and hence not available for all [73, 74]. However, recently, high-throughput DNA sequencing technologies are starting to have an impact on various medical fields and it has become feasible to gather a substantial amount of health information data from patients to be applied for individualized medical treatment [75].

Precision medicine will aid physicians to choose the best treatment and prevention approach for each patient according to their personal health information and their genetic makeup [76]. Certain diseases are associated with and influenced by genetic variations as well as patients’ responses to certain medications. Therefore, the most suitable treatment can be considered to improve the patients’ outcomes. For instance, the finding of hundreds of genetic susceptibility loci in inflammatory bowel disease (IBD) patients contributes to better management of IBD [77]. The development of precision medicine improves our healthcare surroundings by providing precise knowledge of current trends of morbidity and mortality in populations or groups with specific diseases [74].

Various efforts toward precision medicine have been facilitated by many national and international public databases that classify and annotate genomic variation [78], including database for single nucleotide polymorphism (dbSNP) [29], ClinVar [7], and Online Mendelian Inheritance in Man (OMIM) [79]. The basis of precision medicine lies in cataloging genomic variation data of local genomes. Strong local reference databases can make precision medicine meaningful and the inadequacy of local reference data is the biggest challenge in promoting precision medicine in Asia.

Additionally, a decision from expert panels for variant annotation might be one of the challenges in precision medicine. Experts disclose that the disadvantages of personalized medicine are classifiable into 3 recognized issues, which are validity uncertainty, equity issues, and implementation [80]. Therefore, the availability of artificial intelligence (AI) is critical for the implementation of precision medicine. When AI is introduced into precision medicine, organizations can implement precision medicine using their own innovative approach. For instance, AI controls deep learning approaches to defeat the difficulties involved in massive data sets and unstructured data. To illustrate, Gubatan et al. [81] explained the most recent advanced technologies of AI in disease diagnosis, risk prediction, and disease severity evaluation, as well as prediction of clinical decision-making in IBD patients. Again, Jiang et al. [82] developed predictive models learned from historical data for predicting COVID-19 severity. Therefore, we deduce that variant annotation can be implemented into AI to assist in precision medicine in the future.

In clinical settings, AI facilitates clinicians to work more efficiently to make diagnoses more accurate. Additionally, AI helps companies in drug development to implement time and cost-effective measures as well as prevent errors in the system.

## Global initiative toward precision medicine

Globally, there are many initiatives to predict, with a high level of accuracy, which treatment and prevention strategies would be an appropriate choice for any particular disease that may present in a population. A primary challenge for clinical laboratories is to determine, with adequate evidence, the genes that can be associated with clinical findings and patients’ phenotypes. Therefore, ClinGen developed a method to help the community in this effort to assess the strength of evidence for each gene in specific diseases. ClinGen (clinicalgenome.org) is a multi-institutional initiative to standardize gene and variant curation as well as to improve the utility of genomics in medicine. ClinGen developed a method to evaluate the strength of evidence for providing supporting claims for every gene that causes particular diseases. Additionally, this effort was purposely initiated to establish an authoritative central resource for the characterization of clinically relevant genes and variants for precision medicine and future research [32].

ClinGen gene and variant curation is currently organized into 8 Clinical Domain Working Groups (CDWG) to emphasize on curation and standardization efforts in clarification. These CDWG include Cardiovascular, Hearing Loss, Hemostasis or Thrombosis, Hereditary Cancer, Inborn Errors of Metabolism, Neurodevelopmental, Neuromuscular, and RASopathies (mutations in genes of the Ras-MAPK pathway). Each CDWG manages numerous Gene or Variant Curation Expert Panels (GCEPs or VCEPs) performing curation activities on gene sets and diseases within the domain group [83].

As the ACMG–AMP Variant Interpretation Guidelines clarify and specify, ClinGen VCI curates the variants and expert review, and the VCEPs can provide interpreted variant sets to ClinVar and the ClinGen evidence repository (eRepo; erepo.clinicalgenome.org). This step encourages the sharing of data from diagnostic laboratories, creation of consensus interpretations, and development of a collection of gene-specified ACMG–AMP evidence codes, thereby paving the way for eventually ensuring more accurate interpretations for clinical use [75].

However, the critically important factor would also depend on ancestry and population reference data that are supported by previous data, developed through a detailed clinical investigation of affected patients and at-risk family members [84]. As the composition of pathogenic variants differs greatly among human populations, most data focused mainly on populations of European ancestry. In contrast, little is known about pathogenic DNA variants among populations from other parts of the world. This also includes those non-annotated variants that could be pathogenic in the minority of populations [85].

## Conclusion

In the era of precision medicine, variant discovery and annotation are required for diagnosis, prevention, and therapy improvement, based on an individually tailored approach; however, progress is still slow and the application of precision medicine in patients’ treatment still needs enhancement. Despite the abundance of raw data, much genetic variation in the human genome still remains uncharacterized. For precision medicine initiatives to progress, it is crucial that the discovery and annotation pipeline for novel variants also be incorporated during the analysis and interrogation of genomic data. Providing a uniform set of annotation resources for certain genetic diseases will ease comparisons and the interpretation of results. It is hoped that the discovery of novel genomic variants and their annotations would accelerate the development of precision medicine, thus benefitting mankind.
